# Low C-Reactive Protein Levels in a Traditional West-African Population Living in a Malaria Endemic Area

**DOI:** 10.1371/journal.pone.0070076

**Published:** 2013-07-26

**Authors:** Ulrika K. Eriksson, David van Bodegom, Linda May, Anna G. C. Boef, Rudi G. J. Westendorp

**Affiliations:** 1 Department of Gerontology & Geriatrics, Leiden University Medical Center, Leiden, the Netherlands; 2 Leyden Academy on Vitality and Ageing, Leiden, the Netherlands; 3 Department of Parasitology, Leiden University Medical Center, Leiden, the Netherlands; 4 Department of Clinical Epidemiology, Leiden University Medical Center, Leiden, the Netherlands; St. Jude Children’s Research Hospital, United States of America

## Abstract

**Background:**

C-reactive protein (CRP) levels are reported to be elevated in populations of African descent living in affluent environments compared to populations of European ancestry. However, the natural history of CRP levels in populations of African descent living under adverse environments remains largely unknown.

**Methods:**

CRP levels were measured with a high sensitivity assay in 624 apparently healthy individuals who contributed blood as part of a study on innate immune responsiveness in a traditional Ghanaian population living under adverse environmental conditions in a malaria endemic area. As a comparison, we included CRP measurements from 2931 apparently healthy individuals from the Dutch population that were included in the same batch of CRP analyses. Associations between CRP and body mass index (BMI), immune responsiveness, and *P. falciparum* parasitaemia were investigated.

**Results:**

In an age- and sex-adjusted model, CRP levels were 0.54 mg/L lower in the Ghanaian compared to the Dutch cohort (1.52 vs. 0.98 mg/L, p<0.001). When accounting for the substantially higher average BMI in the Dutch compared to the Ghanaians (25.6 vs. 18.4 kg/m^2^) the difference in CRP levels disappeared. BMI associated positively with CRP in the Dutch but not in the Ghanaians. In individuals with an acute phase response, CRP levels were higher in the Ghanaian compared to the Dutch cohort (24.6 vs. 17.3 mg/L, p = 0.04). Levels of CRP were positively related to immune responsiveness and *P. falciparum* parasitaemia (all p<0.001) among Ghanaians.

**Conclusions:**

Our study demonstrates that West-Africans do not exhibit an inherently high inflammatory state. The role of genes, environment and gene-environment interaction in explaining reports of elevated CRP levels in populations of African ancestry when compared to other ethnicities living in affluent environments thus merits further investigation.

## Introduction

The ability to mount adequate immune responses towards pathogens is essential for survival. Infectious diseases have through history constituted the leading cause of death and under pre-industrial conditions most individuals would not survive past their mid-twenties. [1] Exposure to pathogens has thus exerted a high selective pressure on the human genome during evolution. [2] With the introduction of sanitation and modern medicine, life expectancy has increased dramatically in middle- and high-income countries and the causes and timing of death have shifted from communicable diseases in childhood to non-communicable diseases at old age. [3] Meanwhile, aging has been shown to be associated with chronic inflammatory processes, a concept sometimes referred to as “inflamm-aging”. [4] Atherosclerosis, the main contributor to cardiovascular disease (CVD) death, has a prominent pro-inflammatory component. [5] It is therefore possible that, as stated in the antagonistic pleiotropy theory of aging, immune processes that are essential for early-life survival in an adverse environment are associated with detrimental effects in later life [6], [7].

C-reactive protein (CRP) is a component of the innate immune system and belongs to a family of highly conserved acute-phase proteins produced by the liver in response to circulating cytokines, primarily interleukin-6. CRP can increase hundred-fold during the acute phase response (APR) to pathogens. CRP binds various microbes and acts as an activator of the complement system and modulator of the adaptive immune system. In addition, CRP can bind certain Fcγ-receptors and interfere with the binding of IgG antibodies. [8] CRP also correlates with body mass index (BMI) and has been shown to be a risk marker of CVD; [9] elevated serum levels predict coronary heart disease, ischemic stroke, and vascular mortality. [10] While Mendelian randomization studies indicate that the association between CRP and BMI is indeed driven by BMI, [11] CRP only appears to be a marker of CVD-risk and not to be a cause in itself. [12] The role of CRP in malaria is not clear. Studies indicate that elevated levels of CRP are associated with malaria morbidity and mortality [13] but there are also findings that reduced levels of C-reactive protein may be an important pathological mechanism in severe malaria [14].

There are indications of ethnic differences in proneness to inflammation. [15] Organ transplant rejection is higher in Blacks compared to Whites, [16], [17] and allele frequencies of inflammatory genes can differ by race. [18], [19] CRP levels have also been reported to be higher in African descendants compared to whites. [20] It is possible that African descendants have a more pro-inflammatory disposition as a consequence of different evolutionary histories and that CRP is a marker of this pro-inflammatory profile. However, there is very little knowledge on “natural” CRP levels in traditional African populations living in an environment similar to recent evolutionary past. We hypothesized that circulating CRP would be higher in a contemporary, but traditional, Ghanaian population living under adverse environmental conditions in a malaria endemic area but almost devoid of classic CVD risk factors, compared to a representative sample of the Dutch population living under affluent conditions.

## Materials and Methods

### Ethics Statement

Ghana: Informed consent was obtained from all participants. In case of minors, informed consent was obtained from the parents as well as the minor. Since the majority of the study participants were unable to read and write, consent was obtained in the form of a thumb print (by using a stamp pad). This is the same procedure as that used in the local hospital to document consent for an operation or treatment. The entire procedure, including the full text that was to be read by the fieldworkers to the participants in their own language, was approved by the Ethical review committee of the Ghana Health Service. An ethical authorisation was also given by the local chiefs and elders of the research area.

Netherlands: All participants gave written informed consent. The study was approved by the Medical Ethics Committee of the Leiden University Medical Centre, Leiden, the Netherlands.

### Study Population & Data Collection

#### Ghanaian population

The study area is located in the Garu-Tempane district in the Upper East Region in Ghana. The district is underdeveloped with an estimated gross domestic product per capita of less than 100 USD and the vast majority of the inhabitants practice (manually laboured) subsistence agriculture and pastoralism. [21] Infectious diseases constitute the main cause of death both in childhood and adulthood. [22] The region is endemic for malaria (85.5% of apparently healthy individuals being infected with *P. falciparum*), hookworm (31.1% *N. Americanus*), and protozoa (43.8% *G. lamblia*) (own unpublished data). The HIV prevalence of 1.5–2.0% is low compared to other sub-Saharan regions. [23] Arterial disease and diabetes are very uncommon and lipid levels are low. [24] Tobacco is self-grown and beer is (mostly) self-brewed. The custom of chewing kola nut is widespread. Smoking is uncommon in women but could be as prevalent as in Western societies in men. The region has been visited annually since 2002 to collect demographic and biological data.

In 2008, venous blood was collected in the morning from 624 apparently healthy volunteers (aged 8–95 years). Many of these volunteers were primarily selected for a study on the relation between immune responsiveness and fertility (unpublished). Women from different age categories of whom fertility histories were previously collected therefore constitute the majority of the current study sample. Age groups were made in advance and participants were included by going from village to village and house to house and ask all eligible persons at home to participate. If an age group was filled, i.e. the number of participants for that age group was reached, no further inclusions in that age group were made. We excluded persons that were pregnant, had just given birth or were suffering from a current illness. The age range of the final study sample was 8–95 years.

The EDTA plasma fraction was stored at −20°C and transported on dry ice to the Netherlands and then kept at −80°C until analyses. Infection with *P. falciparum*, *N. americanus* and/or *G. lamblia* was determined with PCR in blood or stool [25]–[27] and innate cytokine production *ex-vivo* was measured in whole blood samples after stimulation with LPS and zymosan. [28] Infection determination and innate cytokine production was measured in samples collected in the same bleeding session as the samples used for determination of CRP. Intensity of malaria infection was determined based on the amplification cycle in which the fluorescent signal exceeded the background fluorescence in the real-time PCR, with lower values indicative of higher loads of parasitic DNA. Socio-economic status was assessed with a modified Demographic and Health Survey (DHS) wealth index; [21] drinking water source was assessed on a household level in 2007 through interviews and defined as relatively safe (from borehole) or unsafe (from open well or river). Body mass index (BMI) in kg/m^2^ was calculated based on height and weight measurements performed in various years between 2003 and 2009. BMI data from earlier years were included since the correlation between years of measurement was high (Pearson Correlation = 0.79 for BMI 2003-BMI 2009, p-value 0.02). In total, 322 individuals with a CRP measurement also had information on BMI.

#### Dutch population

The Dutch sample stems from the control sample in the population-based, case-control Multiple Environmental and Genetic Assessment (MEGA) of risk factors for venous thrombosis study which has been described in detail elsewhere. [29], [30] The MEGA study included consecutive patients with a first diagnosis of venous thrombosis selected from the files of the anticoagulation clinics in Amsterdam, Amersfoort, The Hague, Leiden, Rotterdam and Utrecht. Partners of venous thrombosis patients (n = 3796) and a random digit dialling group (n = 3000) were included as control subjects. Controls were eligible for inclusion if between 18 and 70 years old, Dutch speaking, without severe psychiatric problems, and without a recent history of venous thrombosis. Information on ethnic origin was collected by self-reported place of birth of the participants and the place of birth of the parents of the participants. Approximately 90% of all participants were from North or Western European origin. Controls with cancer (n = 499) were excluded from the analyses as were 3362 individuals without a CRP measurement and 4 individuals without information on age. Between 1999 and 2004, blood samples were collected in trisodium citrate tubes and the serum fraction was stored at −80°C until analyses. Height and weight was based on self-report (n = 2851). The age range of the final study sample was 18–70 years.

### Measurements of C-reactive Protein

The samples from Ghana and the Netherlands were analysed simultaneously at the laboratory of clinical chemistry (CKCL) at the Leiden University Medical Center in the second half of 2010. The concentration of CRP was measured with a high-sensitivity immuno-turbidimetric assay (Roche Cobas Integra 800, Roche Diagnostics, Switzerland). The overall coefficient of variation (CV%) was 4.56 and similar in the two study groups. CRP measurements in serum and EDTA-plasma are reported to give similar results in immuno-turbidimetric assays [31].

### Analyses

The chi-squared test was applied to assess differences in frequencies. Student’s t-test was applied for differences in means. CRP values were not normally distributed and were therefore transformed with the natural logarithm. Associations between independent variables and lnCRP were estimated in linear regression models. To present the results of the regression analysis on the original scale instead of the logarithmic scale, regression coefficients were back transformed (i.e. exponentiated). The values can then be interpreted as the percent change in expected geometric mean of CRP per unit increase in the independent variable; e.g. a regression coefficient of 0.282 equals 32.6% (e^0.282^ = 1.326) change in geometric mean CRP. Age- and sex-adjusted geometric mean levels of CRP were estimated using multivariable linear regression. We used PASW (SPSS) Statistics v17 for all analyses.

The two study cohorts differ in both genetic ancestry and current environmental surroundings and it is therefore not possible to disentangle the effects of genes and environment or estimate gene-environment interaction with this study setting.

## Results

**Figure 1:**
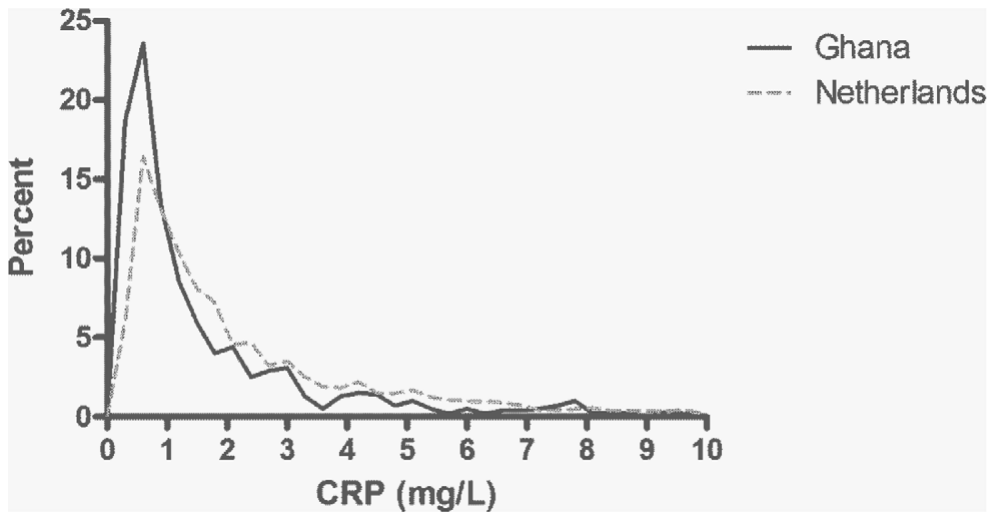
Distribution of circulating C-reactive protein levels in the Ghanaian and Dutch study populations. The graph is restricted to CRP levels up to and including 10 mg/L.

The characteristics of the study samples are depicted in Table 1 and the distribution of CRP in the Ghanaian and Dutch cohorts is depicted in Figure 1. The Ghanaian study sample had significantly lower median and geometric mean levels of CRP compared to the Dutch sample. The Ghanaian cohort had a non-significantly higher proportion of individuals with an acute phase response (defined as CRP≥10 mg/L) compared to the Dutch. Ghanaians with an acute phase response had on average higher CRP levels than the Dutch with an APR (24.6 vs. 17.3 mg/L; p-value 0.04). The distribution of CRP was more skewed toward concentrations between 3 and 10 mg/L, characteristic of systemic low-grade inflammation, in the Dutch cohort.

**Table 1:** Study sample characteristics.

	Ghana	Netherlands	p-value
n	624	2931	
Women, n (%)	463 (74.2)	1539 (52.5)	<0.001
Age (years), mean (SD)	47.3 (20.3)	48.6 (12.4)	0.13
CRP, median (25^th^–75^th^ percentile)	0.8 (0.4–2.2)	1.4 (0.7–3.2)	<0.001
CRP, geometric mean	1.0	1.5	<0.001
Acute phase response*, n (%)	33 (5.3)	121 (4.1)	0.20
Acute phase response*, geometric mean CRP (SD)	24.6 (2.4)	17.3 (1.6)	0.04
Low grade inflammation**, n (%)	79 (12.7)	650 (22.2)	<0.001
BMI, mean (SD)	18.4 (2.5)	25.6 (4.0)	<0.001

CRP: C-reactive protein (mg/L), BMI: body mass index (kg/m^2^),

* CRP≥10 mg/L;

** 3≤CRP<10 mg/L.

CRP concentration associated with age in both study samples with on average 1% higher CRP per year (p-value<0.001). CRP was not associated with sex in the Ghanaian cohort (−0.11 mg/L lower CRP in women vs. men of the same age, p-value 0.38) but Dutch women had on average 0.41 mg/L higher CRP compared to men of the same age sex (p-value<0.001).

**Figure 2:**
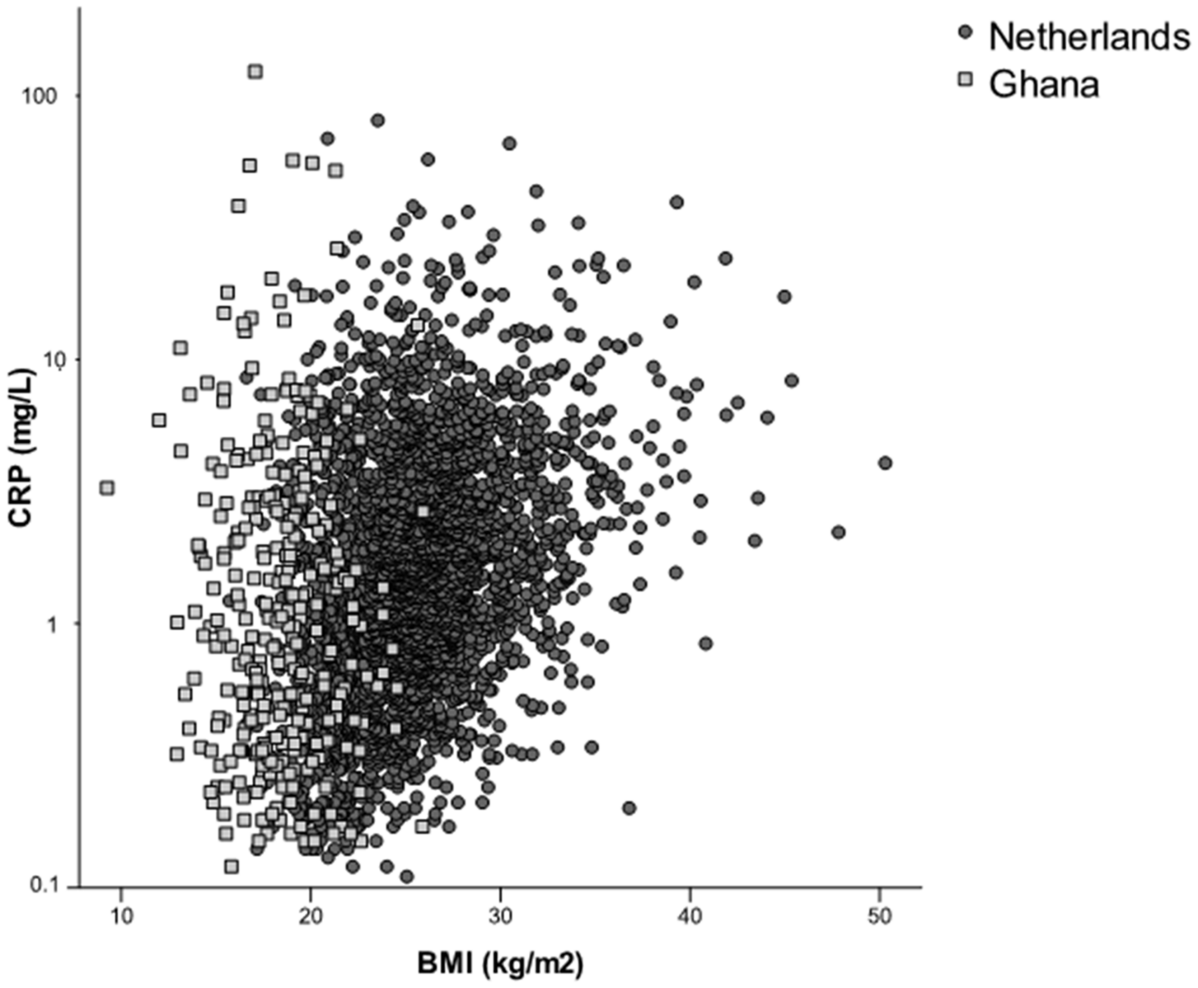
Relationship between BMI and C-reactive protein. Each point represents an individual. Circles represent the Dutch study population; squares the Ghanaian study population. The y-axis is on a logarithmic scale.

The association between BMI and CRP in the two study samples is depicted in Figure 2. Data on BMI was available for 322 individuals in the Ghanaian sample and 2851 in the Dutch sample. There were no significant associations between CRP and BMI in the Ghanaian study sample (2% reduction in CRP with every unit increase in BMI, p-value 0.54) whereas BMI in the Dutch cohort was associated with 9% higher CRP per unit increase in BMI (p-value<0.001). To investigate the effect of BMI on CRP levels in the Dutch cohort for a range of BMI values more similar to those of the Ghanaian sample, the analysis was restricted to the lowest BMI quartile (BMI<22.77). BMI was then no longer associated with higher CRP levels in the Dutch sample.

The variance explained (R^2^) by the predictors age, sex and BMI was 0.14 in the Dutch sample and 0.02 in the Ghanaian.

**Figure 3:**
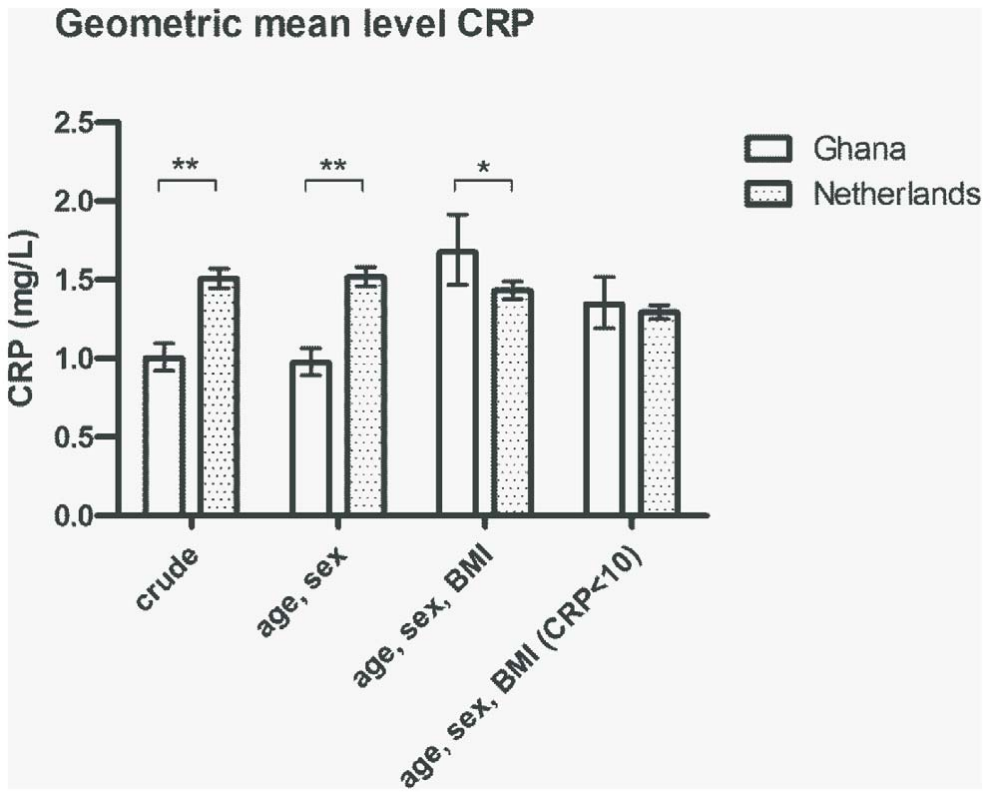
C-reactive protein levels in the Ghanaian and Dutch study populations. Columns indicate geometric mean CRP levels with 95% confidence intervals from general linear models including no (crude) or multiple (age/sex and age/sex/BMI) covariates. The right most columns show the mean levels of CRP for values of CRP below 10 mg/L. *p-value<0.05, **p-value<0.001.

The difference in CRP levels between the two cohorts was estimated with linear regression and is displayed in Figure 3. In the age- and sex- adjusted model, the Ghanaian cohort had lower CRP levels compared to the Dutch cohort (0.98 vs. 1.52 mg/L; p-value<0.001). After adjusting for BMI, the Dutch cohort no longer had higher CRP levels compared to the Ghanaians. To ensure that the changes in estimates after including BMI in the model were not due to differences in study samples (as not all study participants had a value for BMI), the age- and sex-adjusted model was rerun on the subset of individuals with information on BMI but no differences were found. To analyse differences between the Ghanaian and Dutch cohorts for CRP levels in the normal range, the age-, sex- and BMI-adjusted analyses were rerun on the subset of individuals without an acute phase response (i.e. only individuals with CRP<10 mg/L). There were then no significant differences in CRP levels between the Ghanaians and the Dutch.

The effect of infectious and environmental variables on circulating CRP was investigated in the Ghanaian cohort and is depicted in Table 2. Age and LPS/zymosan-stimulated interleukin-10 (IL-10) and tumour necrosis factor α (TNF-α) were significantly associated with CRP in the fully adjusted model. The mean CRP level increased with 32.6% (95% CI 17.2 to 50.1%) per standard deviation increase in innate TNF-α production capacity. Similarly, CRP decreased with 19.7% (9.3 to 28.9) with increased IL-10 production. The proportion of the variance accounted for (R^2^) by the fully adjusted model was 0.069. Higher levels of *P. falciparum* parasitaemia associated with higher circulating geometric mean CRP levels; geometric mean CRP per quartile of *P. falciparum* load was (from low to high): 0.73 mg/L, 0.95 mg/L, 0.97 mg/L and 1.66 mg/L (p for trend<0.001) (age and sex held constant).

**Table 2:** Associations with CRP concentrations in the Ghanaian study sample.

	*Age- and sex-adjusted*		*Fully adjusted*	
	% change in mean CRP	95% CI	% change in mean CRP	95% CI
Age (years)	0.5	0.0 to 1.0	0.7	0.1 to 1.3
Female sex	−10.0	−28.7 to 13.8	−1.6	−23.3 to 26.4
Relative poverty†	13.8	−8.8 to 41.9	16.2	−9.4 to 48.9
Unsafe drinking water‡	23.6	−2.8 to 57.1	16.8	−10.4 to 52.2
Malaria (*P. falciparum*)*	34.3	−0.1 to 80.8	14.8	−16.9 to 58.7
Malaria (*P. falciparum*) load**		<0.001▪		<0.001▪
* quartile mid-low*	29.2	−4.7 to 75.2	36.4	−2.9 to 91.7
* quartile mid-high*	31.9	−3.0 to 79.3	30.9	−7.6 to 85.3
* quartile high*	126.8	65.2 to 211.1	108.1	45.2 to 198.6
Hookworm (*N. americanus*)*	−0.4	−20.9 to 25.6	−2.3	−23.7 to 25.2
Protozoa (*G. lamblia*)*	6.4	−14.5 to 32.3	15.1	−8.8 to 45.4
IL-10	−9.7	−19.6 to 1.3	−19.7	−28.9 to -9.3
TNF-α	24.4	10.7 to 39.7	32.6	17.2 to 50.1

Results are from linear regression models. Per cent change in expected geometric mean CRP (mg/L) per unit increase in the independent variable is equivalent to the absolute value of (1-e^β^)*100. Significant values are indicated by 95% confidence intervals that do not include 0.

The fully adjusted linear regression model included all listed independent variables (malaria load was not included in the analyses of malaria infection and vice versa).

† The poorest half of the population vs. the wealthiest half of the population as estimated by the DHS wealth index.

‡ Unsafe water source (open well or river) vs. safe water source (borehole);

* Infection positivity determined by PCR.

** Load was determined by number of PCR cycles and evaluated in quartiles with the lowest quartile as reference level.

Z-scores of LPS- and zymosan-stimulated levels;

▪ p-value for trend.

## Discussion

We found unexpectedly low circulating CRP levels in a rural, lean, and apparently healthy Ghanaian population living under adverse environmental conditions in a malaria endemic area. In comparison to a European reference population, the Ghanaians had on average 36% lower CRP levels. The difference in CRP levels between the two cohorts disappeared when taking into account the substantially higher BMI of the Dutch compared to the Ghanaian population. BMI was the major factor in explaining the difference in CRP levels between the two populations included in this study; the quarter of the Dutch sample with the lowest BMI had similar CRP levels as the Ghanaian sample and among these individuals the linear association between BMI and CRP disappeared.

There was large variation in CRP levels in the Ghanaian study sample. CRP associated strongest with the production of the pro- and anti-inflammatory cytokines TNF-α and IL-10, as measured in an ex-vivo stimulation assay. This assay reflects both the genetic predisposition to pro- and anti-inflammatory responses as well as on-going sub-clinical infections. [28] Although there was no significant difference in the proportion with an acute phase response in the Dutch and the Ghanaian cohorts, the mean concentration of CRP in those with an APR was higher amongst the Ghanaians compared to the Dutch.

Our study provides new data on CRP levels in a large sample of both men and women in a contemporary West-African population living in an environment similar to recent evolutionary past. However, even in the fully adjusted models, we could only explain 2–7% of the variation in CRP levels in the Ghanaian population. CRP does not have a circadian rhythm and timing of blood sampling would therefore not be a contributor to this variation. [32] The level of CRP does however, increase and decrease rapidly in response to infection and since this cohort is set in a highly infectious environment one plausible contributor to the variation is recent past infection or a recent new infection in latency phase.

A large part of the pathogenic load in tropical Africa is due to one specific parasite, namely *P. falciparum*. The role of CRP in malaria is not clear but CRP levels are known to be highly elevated in acute stages of the disease. [33]–[38] In this study we could also show, with a highly sensitive PCR method and highly sensitive measurements of CRP, that parasite load associates with CRP levels also in asymptomatic individuals. It appears that it is not the occurrence of *P. falciparum* infection per se that leads to elevated CRP levels but the intensity of the infection. CRP has also been proposed to be implicated in the susceptibility to malaria and to provide an explanation to ethnic differences in *P. falciparum* susceptibility [39].

There are some limitations to our study. Although we have shown that West-Africans do not exhibit inherently high circulating CRP levels it is not possible with this study design to conclude on the effect of genes, diet, exercise, smoking, alcohol consumption, or unmeasured infections on CRP levels in West-African populations. It is also not possible to disentangle the effect of genes, environment and gene-environment interaction in explaining observed differences between the Ghanaian and the Dutch study samples. To draw conclusions on the role of genes and environment on the levels of CRP a different study design would be necessary, for example through comparing Ghanaians in Ghana with Ghanaians living under affluent conditions in Europe. Alternatively, a study of Ghanaians in rural and urban environments in Ghana could shed some light on the separate role of genes and the environment.

Two different methods for estimating BMI were used; self-report in the Dutch cohort and physical measurements in the Ghanaian cohort. However, it has previously been shown that there is a high correlation between self-reported and measured BMI and that there are no significant differences in their association with CRP. [40] This should therefore have only a minimal effect on our results.

Studies investigating the effect of ancestry on CRP levels have shown that African Americans have higher levels than White, Asian and Hispanic populations. [41]–[43] Although it appears as if some of the between group variation can be explained by smoking prevalence, BMI, socio-economic status, and different frequencies of polymorphisms in the CRP gene, a large part of the variation in CRP levels cannot be explained by these factors. The heritability of CRP is 27 to 52% which indicates a moderate genetic influence on the variation in CRP levels. [44], [45] African Americans have an estimated mean West-African ancestry of 77% [46] and are thus genetically similar to West-African populations. The affluent environment of modern America is however markedly different from the traditional, adverse conditions of tropical Africa in which the genes were selected for. It has been proposed that common phenotypes in affluent environments, such as obesity and systemic inflammation, can be due to gene-environment interaction, a notion that is indirectly supported by our finding that a West-African population residing in its original environment has low BMI and low CRP [47]–[49].

In conclusion, our study demonstrates that West-Africans do not exhibit an inherently high inflammatory state. The role of genes, environment and gene-environment interaction in explaining reports of elevated CRP levels in populations of African ancestry when compared to other ethnicities living in affluent environments thus merits further investigation.
